# Associations between Maternal Antioxidant Intakes in Pregnancy and Infant Allergic Outcomes 

**DOI:** 10.3390/nu4111747

**Published:** 2012-11-14

**Authors:** Christina E. West, Janet Dunstan, Suzi McCarthy, Jessica Metcalfe, Nina D’Vaz, Suzanne Meldrum, Wendy H. Oddy, Meri K. Tulic, Susan L. Prescott

**Affiliations:** 1 School of Paediatrics and Child Health Research, University of Western Australia, PO Box D184, Princess Margaret Hospital, Perth WA 6001, Australia; Email: christina.west@pediatri.umu.se (C.E.W.); dunstan.jan@gmail.com (J.D.); suzi.mccarthy@health.wa.gov.au (S.M.); jmetcalfe@meddent.uwa.edu.au (J.M.); ndvaz@meddent.uwa.edu.au (N.D.); smeldrum@meddent.uwa.edu.au (S.M.); mtulic@meddent.uwa.edu.au (M.K.T.); 2 Department of Clinical Sciences, Pediatrics, Umeå University, 901 87 Umeå, Sweden; 3 Centre for Child Health Research, University of Western Australia, Telethon Institute for Child Health Research, Perth, PO Box 855, Perth, WA 6872, Australia; Email: wendyo@ichr.uwa.edu.au; 4 EA 6302 Tolerance Immunitaire, Université de Nice Sophia-Antipolis, 06202 Nice, France

**Keywords:** allergy, allergic disease, antioxidant, childhood, copper, diet, dietary intake, eczema, pregnancy, vitamin C

## Abstract

Antioxidant intakes in pregnancy may influence fetal immune programming and the risk of allergic disease. We investigated associations between maternal intakes of β-carotene, vitamin C, vitamin E, copper and zinc, and infant allergic outcomes. Antioxidant intakes of pregnant women (*n* = 420) assessed prospectively by a food frequency questionnaire, were examined in relation to allergic outcomes at 1 year of age (*n* = 300). The main relationships with allergic outcomes were seen with dietary vitamin C and copper. Specifically, higher maternal dietary vitamin C intake was associated with a reduced risk of any diagnosed infant allergic disease and wheeze. After adjustment for potential confounders the relationship with wheeze remained statistically significant. There was also an inverse linear relationship between vitamin C and food allergy. Higher dietary copper intake was associated with reduced risk of eczema, wheeze and any allergic disease. The relationship with wheeze and any allergic disease remained statistically significant in multivariate analysis, and there was also an inverse linear relationship between copper and food allergy. However, these relationships were only seen for nutrients present in food. There were no relationships between β-carotene, vitamin E or zinc and any allergic outcomes. In summary, this study suggests that maternal diet of fresh foods rich in vitamin C is associated with reduced risk of infant wheeze, and that copper intake is associated with reduced risk of several allergic outcomes.

## 1. Introduction

Maternal dietary changes in pregnancy are one of the key environmental factors implicated in the allergy epidemic [1]. Eczema and food allergy are often evident within the first months of life, which highlights the need to investigate the role of early life exposures. As a supply of all nutrients, maternal diet has significant potential to modify the success or failure of immune tolerance and consequently the development of allergic disease in the offspring [2]. Modern, urbanized diets differ in many respects from more traditional diets, with more complex, processed and synthetic foods and less fresh fish, fruits and vegetables. As a consequence, there has been a decline in the intake of many nutrients, including some antioxidants. Declining antioxidant intake is one of a number of dietary changes associated with the steep increase in allergic disease [3]. Earlier epidemiological studies suggested an association between lower intakes of antioxidant rich foods (such as fresh fruits and vegetables) with deficit effects on lung function [4] and increased risk of childhood wheeze [5,6]. Consequently, it has been hypothesized that these epidemiological associations may reflect a protective effect of antioxidant rich diets in the development of asthma [7].

More recently, dietary antioxidants have been implicated in the developmental context of fetal and infant immune programming with consequences for development of allergic disease [8]. A limited number of birth cohort studies have suggested beneficial associations between higher maternal intakes of antioxidants such as vitamin E [9,10,11] and zinc [9] and the development of early childhood wheeze, and asthma [12]. Few studies have investigated the effects of maternal antioxidant intakes and the development of other allergic manifestations than wheeze or asthma [10,11,13] and none has specifically targeted a population at high risk of developing allergic disease.

Therefore, the aim of this study was to prospectively assess the effects of maternal intakes of selected antioxidants (β-carotene, vitamin C, vitamin E, copper and zinc) in pregnancy on early infant allergic outcomes, namely eczema, Immunoglobulin E (IgE)-mediated food allergy, allergic sensitisation and wheeze in a population at high risk of developing allergic disease based on familial association.

## 2. Experimental Section

### 2.1. Overall Study Design

We included 450 mother-infant pairs from a pregnancy cohort, recruited in Perth, Western Australia from 2005 to 2008. The design of this study has previously been described in detail [14]. Briefly, 450 pregnant women with a family history of allergic rhinitis, asthma, eczema, food or other allergy were recruited to participate in a postnatal infant dietary intervention study. This cohort also offered the opportunity to examine the effect of maternal diet in pregnancy (*i.e.*, *prior* to the intervention) on infant clinical outcomes after adjusting for potential confounding factors including the intervention. Briefly, the postnatal randomised trial involved allocation to either 650 mg of fish oil (containing 280 mg docosahexaenoic acid and 110 mg eicosapentaenoic acid) or placebo (olive oil) for the first six months of life. Women were ineligible if they were smokers, had pre-existing medical conditions other than allergy, pregnancy complications, delivered pre-term (gestation <36 weeks), ate >3 meals of fish per week or used fish oil supplementation >1000 mg/day during pregnancy. The study was approved by the Princess Margaret Hospital Research Ethics Committee and the participants provided written informed consent. The study was registered at the Australian New Zealand Trials Registry; ACTRN12606000281594. Data from a semi-quantitative food frequency questionnaire (SQFFQ) completed at the last trimester of pregnancy (after 28 weeks gestation) and infant clinical outcome measures at 12 months were available in 144 and 156 mother-infant pairs from the fish oil and placebo groups, respectively (*p* > 0.05). Thus, 300 mother-infant pairs were available for the purpose of this study. 

As discussed elsewhere, infant fish oil supplementation had no effect on the clinical outcome measures, neither in the whole study population [14] nor in the 300 mother-infant pairs available for the purpose of this study. 

### 2.2. Demographic Characteristics and Dietary Assessment

Parental demographic information and health history information were collected by an interview and a questionnaire at the recruitment visit. Data on infant characteristics and health history were collected from detailed questions on the 12 month questionnaire [14]. 

The semi-quantitative food frequency questionnaire (SQFFQ) was administered to all pregnant mothers at the recruitment visit. The SQFFQ was developed and analysed by the Commonwealth Scientific and Industrial Research Organisation (CSIRO), Adelaide, Australia. It provides information on the reported frequency of 212 individual foods, mixed foods and beverages over the preceding month. Reported vitamin and mineral supplement intakes (brand, dose and frequency) was converted into daily β-carotene, vitamin C, E, zinc and copper intakes, using dosage information provided at the packaging.

### 2.3. Maternal Allergy Classifications

Maternal allergy was defined as a history of doctor diagnosed allergy (including asthma, allergic rhinitis, eczema, food or other allergy) [14]. Maternal atopy was defined as a positive skin prick test (SPT) performed at the recruitment visit to at least one allergen (dog, cat, rye, feathers, house dust mite, southern grasses, mold or cockroach; Hollister-Stier Laboratories, Spokane, WA, USA). A wheal diameter of 3 mm was considered positive.

### 2.4. Infant Allergic Outcomes at 12 Months of Age

The main clinical outcome measures were the prevalence of eczema, IgE-mediated food allergy, and allergic sensitisation. Infants were clinically evaluated at 12 months of age, which included a detailed history and examination. A diagnosis of eczema was made in infants with typical skin lesions responsive to topical steroids. The severity of eczema was determined using the severity score of atopic dermatitis (SCORAD) index as previously described [15]. IgE-mediated food allergy was defined as a history of immediate (within 60 min) symptoms after contact with and/or ingestion and a positive SPT to the offending food. Symptoms of acute food allergy included skin reactions (hives, rash or swelling) and/or respiratory symptoms (cough, wheeze, stridor) and/or gastrointestinal symptoms (abdominal pain, vomiting, loose stools) and/or cardiovascular symptoms (collapse). Information was also collected on respiratory symptoms, although the limitations of this data at this early age are well recognized. Asthma was defined as physician diagnosed asthma. If the infant had a diagnosis of eczema, IgE-mediated food allergy (as described above) or asthma they were classified as having allergic disease.

Infants had an SPT at 12 months of age using common allergen extracts (milk, egg, peanut, rye, house dust mite, cat, grass, mold; Hollister-Stier Laboratories, Spokane, WA, USA) and whole egg, as well as histamine as a positive control and glycerine as a negative control. A wheal diameter of ≥2 mm was considered positive. Allergic sensitisation was defined as at least one positive SPT performed at the 12 months visit to at least one allergen.

### 2.5. Statistical Analysis

Intakes of dietary antioxidants were energy-adjusted according to the nutrient residual method [16] and divided into quartiles based on the distribution of the nutrient residuals in 300 subjects. Further, to take into account the total intake of the antioxidant in question, intakes from foods plus vitamin and mineral supplementation were added, energy-adjusted and divided into quartiles based on their distribution. Logistic regression analysis was applied to estimate the crude odds ratios and their 95% confidence intervals of eczema, food allergy, wheeze, sensitisation and diagnosis of any allergic disease with the lowest quartile as a reference when we assessed both dietary and total antioxidant intakes.

We performed bivariate analyses to determine the non-nutrient characteristics associated with the clinical outcomes by using logistic regression. Variables that were significantly associated with any of these outcomes were included in the multivariate analyses. We identified maternal parity (number of previous deliveries), paternal family history of allergic disease (yes/no), delivery mode (labour or non-labour), birth weight and exposure to furred pets (yes/no) as variables to be included in multiple logistic regression analysis. As noted above, the postnatal intervention did not have a significant effect on allergic outcomes, and did not alter the findings of the present study when included in the analysis as a dichotomous variable (intervention/no intervention). Differences were regarded as significant at the level *p* < 0.05. Statistical analyses were performed using SPSS software version 17.0 for Windows [17].

## 3. Results

### 3.1. Population Characteristics

The demographic characteristics of the 300 mother-infant pairs with data on both dietary intakes in pregnancy and infant clinical outcome measures at 12 months of age are displayed in Table 1. In the majority of pregnant women dietary intakes of antioxidants well met the recommended daily intake (RDI) except for vitamin E and zinc (Table 2) [18]. Infant allergic outcomes are displayed in Table 3. There was no difference in the prevalence of any of the clinical outcome measures at 12 months of age between the fish oil and placebo groups (data not shown).

**Table 1:** Population characteristics of 300 mother-infant pairs.

Characteristics	Number analyzed	Mean (S.D.) or *n* (%)
*Mothers at recruitment*		
Maternal age (years)	300	33.5 (1.3)
Gestation at recruitment (weeks)	300	36.6 (1.4)
Completed 12 years of education	248	224 (90.3)
Maternal history of asthma	300	153 (51.0)
Maternal history of eczema	300	125 (41.7)
Maternal history of allergic rhinitis	300	223 (74.3)
Maternal history of food allergy	300	66 (22.0)
Maternal sensitisation at recruitment	300	290 (96.7)
Vitamin and/or mineral supplementation	300	273 (91.0)
*Paternal characteristics*		
Paternal age (years)	293	35.9 (5.3)
Completed 12 years of education	241	196 (81.3)
Paternal history of asthma	296	58 (19.6)
Paternal history of eczema	296	51 (17.2)
Paternal history of allergic rhinitis	296	117 (39.5)
Paternal history of food allergy	296	28 (9.5)
*Environmental exposures*		
Partner smokes	299	30 (10.0)
Pets in the house	298	184 (61.7)
*Infant characteristics*		
Infant sex (% male)	300	160 (53.3)
Infant birth weight (g)	298	3484 (427)
Infant birth length (cm)	297	50.5 (2.4)
Labour	297	218 (73.4)
Caesarean delivery (planned/emergency)	297	115 (38.7)
Gestational age (weeks)	300	39.3 (1.2)
Season when infant was born	300	
January–March		79 (26.3)
April–June		83 (27.7)
July–September		86 (28.7)
October–December		52 (17.3)
Older siblings (% 1 or more)	300	158 (52.7)
Ever breastfed	300	292 (97.3)
Ever formula-fed	300	239 (79.7)
Introduction of solid foods (months)	299	5.6 (0.9)
*Infants at 1 year*		
Still breastfed	299	107 (35.8)
Attending day-care or playgroup	292	240 (82.2)

**Table 2:** Distribution of daily maternal dietary and total intakes of antioxidants in pregnancy.

Variable	Mean (S.D.)	Median (25th–75th percentile)	RDI ^1^
Total energy (kJ/day)	8830 (2573)	8419 (7043–10354)	-
β-carotene (μg/day) ^2^	4687 (2118)	4391(3291–5916)	-
β-carotene ^3^	5025 (2599)	4547 (3331–5999)	
Vitamin C (mg/day) ^2^	201 (98)	193 (130–255)	60
Vitamin C ^3^	337 (239)	274 (188–399)	
Vitamin E (mg/day) ^2^	8.6 (4.0)	7.7 (6.0–10.3)	7.0
Vitamin E ^3^	17.4 (15.3)	13.8 (9.6–20.8)	
Zinc (mg/day) ^2^	11.8 (3.8)	11.2 (9.1–13.6)	11.0
Zinc ^3^	19.7 (8.0)	18.9 (13.7–25.9)	
Copper (mg/day) ^2^	2.0 (0.6)	2.0 (1.6–2.4)	1.3 ^4^
Copper ^3^	2.3 (0.8)	2.2 (1.7–2.8)	

^1^ RDI refers to Recommended Dietary Intake according to the Nutrient Reference Values for Australia and New Zealand in pregnancy (aged 19–50 years), and are displayed when available; ^2^ Dietary intakes; ^3^ Total intakes from diet and supplement; ^4^ This refers to the Adequate Intake (AI) that is used when an RDI cannot be determined.

**Table 3:** Allergic outcomes at 12 months of age.

Allergic outcome	Number analyzed	*n* (%)
Allergic disease	300	118 (39.3)
Eczema	294	118 (40.1)
Food allergy	294	39 (13.3)
Any wheeze	299	85 (28.4)
Asthma	300	0 (0)
SPT^1^ positive	295	85 (28.8)
Egg SPT positive	293	67 (22.9)
Peanut SPT positive	293	32 (10.9)
Milk SPT positive	294	10 (3.4)
HDM SPT positive	293	19 (6.5)
Cat SPT positive	293	13 (4.4)
Grasses SPT positive	293	5 (1.7)

^1^ SPT: skin prick test.

### 3.2. Effects of Maternal Dietary Antioxidant Intakes on the Development of Allergic Disease

First, we analysed the relationship between maternal dietary antioxidant intakes and infant allergic outcomes (Table 4). This was to examine the effects of foods separate to any antioxidants derived from maternal supplements in pregnancy.

**Table 4:** ORs and 95% CIs for 12 month allergic outcomes by quartiles of maternal dietary antioxidant intake in pregnancy in 300 mother-infant pairs ^1^.

	Eczema		Food Allergy		Wheeze			Sensitisation		Any Allergic disease	
	Crude OR	Adjusted OR ^2^	Crude OR	Adjusted OR ^2^		Crude OR	Adjusted OR ^3^	Crude OR	Adjusted OR ^4^	Crude OR	Adjusted OR ^2^
β-carotene											
Q1	1.00	1.00	1.00	1.00		1.00	1.00	1.00	1.00	1.00	1.00
Q2	0.88 (0.46–1.71)	0.54 (0.25–1.17)	0.66 (0.24–1.84)	0.40 (0.12–1.32)		0.61 (0.29–1.28)	0.54 (0.23–1.22)	0.81 (0.39–1.69)	0.54 (0.23–1.22)	0.80 (0.41–1.54)	0.51 (0.24–1.11)
Q3	1.03 (0.53–2.00)	0.76 (0.36–1.64)	1.57 (0.66–3.82)	1.16 (0.43–3.11)		1.30 (0.66–2.57)	1.09 (0.70–2.88)	1.31 (0.66–2.63)	1.09 (0.51–2.32)	1.12 (0.58–2.13)	1.02 (0.49–2.14)
Q4	0.85 (0.44–1.66)	0.67 (0.31–1.43)	0.78 (0.29–2.09)	0.38 (0.11–1.27)		0.77 (0.38–1.57)	0.73 (0.33–1.61)	0.92 (0.45–1.89)	0.73 (0.33–1.61)	0.80 (0.41–1.54)	0.68 (0.32–1.43)
*P*_trend_	0.9	0.5	0.3	0.2		0.2	0.2	0.6	0.3	0.7	0.2
Vitamin C											
Q1	1.00	1.00	1.00	1.00		1.00	1.00	1.00	1.00	1.00	1.00
Q2	1.01 (0.52–1.94)	0.87 (0.41–1.86)	**0.26 (0.08–0.85)**	**0.22 (0.06–0.78)**		0.98 (0.49–1.95)	1.11 (0.51–2.40)	0.57 (0.27–1.21)	**0.22 (0.13–0.78)**	0.68 (0.36–1.31)	0.62 (0.30–1.32)
Q3	0.88 (0.46–1.70)	1.07 (0.50–2.31)	1.06 (0.46–2.44)	0.75 (0.27–2.06)		1.04 (0.60–2.52)	1.33 (0.61–2.90)	1.16 (0.58–2.32)	0.83 (0.37–1.85)	0.76 (0.40–1.46)	0.78 (0.37–1.66)
Q4	0.69 (0.35–1.35)	0.72 (0.33–1.57)	0.54 (0.21–1.40)	0.46 (0.16–1.36)		**0.40 (0.18–0.87)**	**0.40 (0.16–0.98)**	1.03 (0.51–2.07)	0.76 (0.34–1.67)	**0.48 (0.25–0.93)**	0.56 (0.28–1.26)
*P*_trend_	0.7	0.8	0.07	0.1		0.06	0.06	0.3	0.07	0.2	0.5
Vitamin E											
Q1	1.00	1.00	1.00	1.00		1.00	1.00	1.00	1.00	1.00	1.00
Q2	0.98 (0.51–1.89)	1.02 (0.47–2.22)	1.00 (0.40–2.48)	0.96 (0.32–2.84)		0.84 (0.41–1.70)	0.89 (0.41–1.93)	0.78 (0.39–1.56)	0.69 (0.31–1.55)	1.18 (0.62–2.25)	1.21(0.56–2.60)
Q3	0.73 (0.38–1.43)	0.93 (0.43–2.03)	0.78 (0.30–2.01)	0.86 (0.29–2.54)		0.71 (0.35–1.47)	0.72 (0.33–1.41)	0.68 (0.33–1.58)	0.57 (0.25–1.28)	0.55 (0.28–1.09)	0.82 (0.37–1.78)
Q4	1.18 (0.61–2.28)	1.42 (0.68–2.98)	0.68 (0.26–1.81)	0.57 (0.19–1.72)		1.06 (0.53–2.12)	0.77 (0.36–1.66)	0.74 (0.37–1.50)	0.64 (0.29–1.41)	1.06 (0.55–2.02)	1.40 (0.68–2.91)
*P*_trend_	0.6	0.7	0.8	0.8		0.7	0.9	0.7	0.5	0.1	0.5
Copper											
Q1	1.00	1.00	1.00	1.00		1.00	1.00	1.00	1.00	1.00	1.00
Q2	**0.42 (0.22–0.83)**	0.57 (0.27–1.23)	0.62 (0.25–1.55)	0.60 (0.22–1.60)		0.54 (0.27–1.10)	**0.40 (0.18–0.91)**	0.63 (0.31–1.31)	0.40 (0.18–0.91)	**0.41 (0.21–0.80)**	**0.40 (0.19–0.87)**
Q3	**0.40 (0.20–0.78)**	**0.31 (0.14–0.70)**	0.70 (0.28–1.71)	0.40 (0.13–1.22)		0.84 (0.43–1.64)	0.89 (0.42–1.89)	0.98 (0.49–1.95)	0.59 (0.27–1.30)	**0.41 (0.21–0.80)**	**0.34 (0.15–0.74)**
Q4	0.60 (0.31–1.15)	0.58 (0.28–1.21)	0.46 (0.17–1.23)	**0.38 (0.11–0.95)**		**0.39 (0.18–0.81)**	**0.31 (0.13–0.72)**	0.79 (0.39–1.60)	0.64 (0.30–0.39)	0.58 (0.31–1.12)	0.57 (0.28–1.19)
*P*_trend_	0.03	0.05	0.5	0.2		0.05	**0.01**	0.6	0.2	0.03	0.03
Zinc											
Q1	1.00	1.00	1.00	1.00		1.00	1.00	1.00	1.00	1.00	1.00
Q2	1.32 (0.68–2.56)	1.44 (0.67–3.10)	0.86 (0.31–2.37)	0.67 (0.22–2.03)		0.90 (0.45–1.80)	0.77 (0.35–1.66)	0.81 (0.39–1.69)	0.70 (0.32–1.57)	1.26 (0.65–2.46)	1.37 (0.60–2.77)
Q3	1.17 (0.60–2.29)	1.22 (0.56–2.66)	1.40 (0.55–3.56)	1.28 (0.46–3.53)		1.00 (0.50–1.99)	0.91 (0.42–1.98)	1.29 (0.64–2.59)	1.16 (0.53–2.54)	1.26 (0.65–2.46)	1.47 (0.68–3.19)
Q4	1.64 (0.85–3.18)	1.73 (0.80–3.73)	1.11 (0.42–2.92)	0.52 (0.16–1.73)		0.53 (0.25–1.12)	0.47 (0.20–1.08)	1.02 (0.50–2.09)	0.87 (0.39–1.96)	1.85 (0.95–3.57)	1.85 (0.86–3.97)
*P*_trend_	0.5	0.5	0.8	0.4		0.3	0.4	0.7	0.6	0.3	0.5

^1^ Calorie-adjusted nutrients are in quartiles of intake, with the first quartile being the lowest; ^2^ Maternal education, paternal history of allergic disease, birth weight and exposure to furred pets at home were included in the multiple logistic regression model; ^3^ Maternal education and delivery method were included in the multiple logistic regression model; ^4^ Maternal education and parity were included in the multiple logistic regression model; Bold font indicates statistically significant relationship.

For “any allergic disease” higher maternal dietary vitamin C intake was associated with a reduced risk of diagnosed allergic disease at 1 year for the highest *versus* the lowest quartile (the crude odds ratio [OR] = 0.48, 95% CI 0.25–0.93), although the inverse linear trend and the adjusted OR were not statistically significant (Table 4). Higher maternal dietary vitamin C intake was also associated with a reduced risk of wheeze (highest *versus* the lowest quartile crude OR 0.40; 95% CI 0.18–0.87), with the inverse linear trend approaching significance (*p*_trend_ = 0.06). These relationships persisted after adjusting for potential confounders (Table 4). There were also relationships between vitamin C intake and both food allergy (*p*_trend_ = 0.07) and sensitisation (*p*_trend_ = 0.07) although neither of these was statistically significant.

There were also inverse relationships between maternal dietary copper intake and a number of infant allergic outcomes, including eczema (*p*_trend_ = 0.03), wheeze (*p_t_*_rend_ = 0.05) and any allergic disease (*p*_trend_ = 0.03) as noted on Table 4. After adjusting for potential confounding factors these inverse linear trends were still evident (eczema *p*_trend_ = 0.05, wheeze *p*_trend_ = 0.01 and any allergic disease *p*_trend_ = 0.03). Comparing quartiles for copper intake, the risk of wheeze was significantly reduced in the highest *versus* the lowest quartile (OR 0.39; 95% CI 0.18–0.81) and remained statistically significant after adjusting for potential confounders in the multivariate model (aOR 0.31; 95% CI 0.13–0.72). The relationship between copper intakes and food allergy also became statistically significant in the multivariate model (highest *versus* the lowest quartile aOR 0.38 (95% CI 0.11–0.95).

No statistically significant exposure-response associations were observed between maternal dietary intakes of β-carotene, vitamin E or zinc and the risk of developing early allergic outcomes (Table 4). Since many mothers did not meet the RDI for vitamin E and Zinc [18], we investigated associations between vitamin E and zinc intakes dichotomized as exposure at the minimum RDI (yes/no) and allergic outcomes. We observed a trend towards an inverse association between dietary zinc intakes and wheeze (crude OR 0.64; 95% CI 0.39–1.06), (*p*_trend_ = 0.08). However, when adjusting for potential confounders, the inverse trend was not statistically significant (aOR 0.74, 0.42–1.31), (*p*_trend_ = 0.3). No other statistically significant exposure-response associations became evident (data not shown).

### 3.3. Effects of Total Maternal Antioxidant Intakes on the Development of Allergic Disease

Next we examined the same relationships in relation to antioxidants derived from *both* the maternal diet and from supplements. The majority of the mothers consumed some form of vitamin and mineral supplementation (most commonly in the form of a multivitamin supplement) during pregnancy; with no differences according to the postnatal randomized intervention (*p* = 0.4). In summary, there were no statistically significant associations observed between total intake of specific antioxidants (β-carotene, vitamin C, E, zinc and copper) in pregnancy and the risk of developing eczema, food allergy, wheeze, sensitisation or any allergic disease neither in the crude nor adjusted analyses (data not shown). There were also no relationships between antioxidants specifically derived from supplements (as opposed to background diet) and allergic outcomes (data not shown).

## 4. Discussion

In this study, there were some inverse (“protective”) relationships between antioxidant intakes in pregnancy and infant allergic outcomes, but this was only evident for selected antioxidant factors (vitamin C and copper) and only seen in relation to dietary nutrients and not for dietary supplements *per se*. Specifically, higher maternal dietary copper intake was associated with reduced risk of wheeze and development of any early allergic disease in infants at high risk due to family allergy history. Furthermore, maternal dietary vitamin C intake in pregnancy appeared protective against development of wheeze in the first year of life. As indicated, there were no associations between allergic outcomes and total intakes of copper, vitamin C or any other antioxidants when we also took maternal vitamin and mineral supplementation into account. This could be an indication that other qualities of maternal diet and not only the specific antioxidant in question may provide beneficial effects on immune function and disease susceptibility.

While there are relatively few studies to examine copper in this context, there are some previous observations that support a role for this element in immunomodulation. Copper requirements are increased in pregnancy and suboptimal supply may have adverse effects on developing tissues and organ systems including the lung, skin and immune system [19]. Compared with optimal copper intake, a diet marginally low in copper to pregnant rodents led to reduced splenic T cell proliferation as demonstrated by lower mitogen induced Interleukin-2 (IL-2) production in their offspring [20]. In humans, the immune effects of copper deficiency in pregnancy are largely unknown. In the large Project Viva birth-cohort study, a higher total copper intake in pregnancy was associated with reduced wheeze at 2 years in the univariate model, although there was no association when adjusting for confounders [9]. Compared to dietary copper intake levels in that study, levels in the present study are marginally higher. However, the two studies are not necessarily comparable as we studied a high allergy risk population. Our findings suggest that optimal dietary copper intakes specifically in infants at high risk of developing the allergic phenotype may be favourable, although this must be further explored.

The potential protective effects of vitamin C and antioxidant rich foods on allergic disease have been noted in a number of previous studies, including links between higher intakes of antioxidant rich foods, better pulmonary function and reduced risk of wheeze [21]. This is consistent with the findings in the present study that suggest a reduced risk of wheeze with higher maternal dietary vitamin C intake, although the inverse linear trend fell short of the significance level. However, the limitations of assessing wheeze and asthma at this age are well recognized, and results should be interpreted cautiously. There is a sigmoidal association between vitamin C intake and plasma concentration of vitamin C. Levine *et al.* [22] demonstrated that plasma concentrations increased steeply above intakes of 30 mg daily and that the steep portion of the plasma concentration curve occurred with a daily dose of vitamin C of between 30 and 100 mg. Complete saturation occurred at 1000 mg daily. In the present study, the majority of mothers reported dietary vitamin C intakes above the RDI of 60 mg vitamin C daily, thereby well meeting the expected needs [18]. Therefore, we speculate that there was no additional benefit when assessing the relationship between total intakes (diet plus supplements) in relation to development of allergic disease. By comparison, Miyake *et al.* [11] reported an inverse relationship between maternal intake of citrus fruits, rich in vitamin C, and reduced risk of wheeze at 2 years. This negative association between intakes of fruits and asthma and asthma-like symptoms was also demonstrated in a meta-analysis including 22 (cohort, cross-sectional and case-control) studies [23]. With a more limited dataset available, a meta-analysis on two published birth cohort studies [9,10] observed no association between maternal vitamin C intake in pregnancy and wheeze at 2 years of age [23]. There is a paucity of intervention studies with vitamin C in pregnancy for prevention of allergic disease. Although not designed for allergy prevention, Greenough *et al.* [24] observed no effect on respiratory outcomes up to 2 years of age in children whose mothers at high risk of preeclampsia were supplemented with high dose vitamins C and E in pregnancy, compared to placebo. 

We could not confirm previous findings that reduced maternal vitamin E intakes were associated with increased risk of developing childhood wheeze [9,10,11,12] despite the fact that almost 50% of pregnant mothers did not meet the RDI when intakes from diet alone were taken into account, and 20% of the mothers did not meet the RDI when total vitamin E intakes were analysed [18]. Finally, we observed no statistically significant exposure-response associations between maternal dietary intakes of β-carotene or zinc, and the risk of early allergic outcomes. This is in contrast to the meta-analysis by Nurmatov *et al.* [23] that suggested a possible effect of zinc in pregnancy and the development of asthma. Again, as we specifically included a population at high risk of developing the allergic phenotype, studies included in the meta-analysis and the present study are not directly comparable. Further, assessing preventive effects on asthma at this young age is controversial as much wheeze is infection-induced at this age.

This study has several strengths. The prospective design limits the risk of recall bias. In previous studies [9,10,11,13] the diagnosis of allergic disease has relied on data from questionnaires and/or evidence of sensitisation whereas in the present study, the diagnosis of allergic disease relies on both clinical assessment and evidence of allergic sensitisation. Information on a number of potential confounders such as dietary exposures, including breastfeeding duration, and other environmental exposures were collected prospectively. Their possible association with the clinical outcomes were examined, and variables that were significantly associated with any of these outcomes were then included in the multivariate analyses. However, there are also limitations of the study. As with all observational studies assessing effects of specific antioxidants there is potential confounding related to the issue of concomitant intakes of other protective nutrients. It has been argued that assessment of the effects of specific nutrients individually may be over-simplistic. An optional approach is to examine the effects of changing composite patterns of specific nutrient intakes [2]. “Dietary patterns” such as the Mediterranean diet has been suggested to be protective in the development of allergic disease [25,26], largely due to dietary effects in pregnancy [26].

## 5. Conclusions

In summary, this study suggests an association between maternal consumption of foods rich in some antioxidant factors, namely vitamin C and copper, and reduced risk of allergic outcomes in infancy in a population predisposed to allergic disease. However, there were no significant effects of any other antioxidants on these outcomes. The protective effects of fresh foods rich in vitamin C on wheeze is consistent with previous studies, and it is notable that we did not observe any effect of vitamin supplements on any allergic outcomes. Further studies are needed to assess the effects of both specific antioxidants and dietary patterns in pregnancy, with a particular focus on the complex composite effects of diet and the effects of fresh foods rather than isolated supplemental nutrients. 
